# Comparative Evaluation of the Marginal Fit of Zirconia and Polyetheretherketone Copings Fabricated Using Computer-Aided Design and Computer-Aided Manufacturing (CAD/CAM) Technology: A Systematic Review and Meta-Analysis

**DOI:** 10.7759/cureus.96664

**Published:** 2025-11-12

**Authors:** Radha L Harimkar, Niraja Jaiswal, Arti P Gangurde, Manish Chauhan, Sahil Shaikh, Sanjana Bhosale

**Affiliations:** 1 Department of Prosthodontics and Crown and Bridge, Government Dental College and Hospital, Mumbai, IND

**Keywords:** cad/cam milling, fixed dental prosthesis, marginal adaptation, polymer-based restorations, prosthesis

## Abstract

Accurate marginal adaptation is essential for the clinical longevity of fixed dental restorations. Zirconia and polyetheretherketone (PEEK) have emerged as popular computer-aided design and computer-aided manufacturing (CAD/CAM) materials for dental copings, but limited evidence exists directly comparing their marginal fit. This systematic review aimed to critically evaluate and compare the marginal fit of zirconia and PEEK copings fabricated using CAD/CAM systems. This review protocol was prospectively registered in PROSPERO (CRD42024604881). An electronic literature search was conducted in PubMed, SCOPUS, Web of Science, Google Scholar, Embase, and EBSCOHost to identify studies published between 2016 and 2025. Eligible studies included in vitro experiments comparing the marginal fit of zirconia and PEEK copings using quantitative measurements. Data extraction and risk of bias assessment were independently performed by two reviewers using a standardized form and the QUIN tool. A meta-analysis was conducted using Review Manager software where appropriate, and heterogeneity was assessed using I² statistics. Eight studies met the inclusion criteria. Sample sizes ranged from 18 to 40, using extracted teeth or typodont/resin models. Zirconia demonstrated superior marginal fit in six of eight studies, with mean gap values ranging from 17 to 60 µm. Meta-analysis of five studies (n=126 samples) showed a standardized mean difference (SMD) of 1.72 µm (95% CI: -0.46 to 3.90 µm; p = 0.12) favoring zirconia, though the result was not statistically significant. High heterogeneity (I² = 95%) was observed. Both zirconia and PEEK copings exhibit clinically acceptable marginal adaptation. Zirconia generally shows better consistency, while PEEK remains a promising alternative under optimized fabrication conditions. Further standardized in vitro and clinical studies with aging simulations are warranted to guide evidence-based material selection.

## Introduction and background

Dental copings serve as the foundational substructure of fixed prosthodontic restorations, providing essential support to the overlying esthetic and functional layers while ensuring mechanical durability and biological harmony with surrounding oral tissues [1,2]. Historically, a diverse array of materials, including metals, ceramics, and polymers, has been used in coping fabrication, each offering distinct advantages tailored to specific clinical contexts [3]. Metal alloys, such as gold and nickel-chromium, have long dominated the prosthodontic landscape due to their strength, malleability, and excellent marginal adaptation [4,5]. However, a growing emphasis on esthetic outcomes and biocompatibility has catalyzed a shift toward ceramics and high-performance polymers over the past few decades [6,7]. Among the contemporary alternatives, ceramics and zirconia have gained prominence, each presenting unique mechanical profiles and challenges in clinical use [8]. Zirconia, a high-strength ceramic, is well-regarded for its superior mechanical behavior, biocompatibility, and favorable esthetic characteristics [9]. Its transformation-toughening mechanism enhances resistance to crack propagation, making it particularly suitable for high-stress bearing areas of the oral cavity [10]. The advent of computer-aided design and computer-aided manufacturing (CAD/CAM) technologies has further optimized the precision and reproducibility of zirconia restorations, improving their clinical consistency [11]. Nevertheless, despite these advantages, zirconia’s inherent brittleness and dimensional changes during sintering can lead to discrepancies in marginal fit, an aspect critical to long-term prosthetic success [12].

Conversely, polyethyletherketone (PEEK) has emerged as a compelling alternative in prosthodontics, derived from its roots in orthopedic applications [13]. As a high-performance thermoplastic polymer, PEEK exhibits favorable characteristics, such as light weight, high resilience, and a modulus of elasticity that closely mimics that of bone, enabling efficient stress distribution and shock absorption [14]. It also demonstrates resistance to plaque accumulation and chemical degradation, making it suitable for long-term intraoral application [15]. However, the challenge lies in achieving precise marginal adaptation with PEEK, as its material properties and milling behavior differ markedly from those of ceramics such as zirconia [16].

Marginal fit plays a pivotal role in the overall success of prosthodontic restorations [17]. Accurate marginal adaptation prevents the ingress of fluids and bacteria, thereby reducing the risk of secondary caries, marginal inflammation, and eventual prosthesis failure [18,19]. Even minor marginal discrepancies can significantly compromise the structural and biological integrity of restorations, resulting in reduced clinical longevity and patient satisfaction [20]. The ability to achieve optimal marginal fit depends on several interacting factors, including material properties, fabrication protocols, and cementation techniques [21,22]. Given the increasing use of zirconia and PEEK in clinical practice, understanding their comparative performance with respect to marginal adaptation is of critical importance for evidence-based material selection.

The integration of digital workflows has transformed the evaluation of marginal fit [23]. CAD/CAM systems now enable enhanced control over coping design and milling parameters, minimizing operator-dependent variability and improving standardization [24-26]. Additionally, modern measurement techniques such as micro-computed tomography, stereomicroscopy, silicone replica techniques, and digital scanning provide high-resolution insights into marginal gaps and restoration fit [27]. These advancements have facilitated more accurate and reproducible comparisons between materials such as zirconia and PEEK, thus contributing to the refinement of clinical workflows and laboratory protocols [28].

Despite these technological strides, there remains a paucity of consolidated evidence directly comparing the marginal adaptation of zirconia and PEEK copings [29]. While several studies have independently investigated the fit of these materials, the heterogeneity in methodologies, measurement tools, and fabrication parameters necessitates a systematic synthesis of findings. Such a synthesis would bridge existing knowledge gaps and inform clinical decision-making in scenarios where marginal accuracy is paramount to prosthetic longevity and patient outcomes. In light of these considerations, the present systematic review was undertaken to critically assess and compare the marginal fit of zirconia and PEEK copings across in vitro experimental studies.

## Review

Methodology

The present systematic review was conducted in accordance with the Preferred Reporting Items for Systematic Reviews and Meta-Analyses (PRISMA) guidelines [30]. The protocol was prospectively registered with the International Prospective Register of Systematic Reviews (PROSPERO) under the registration ID CRD42024604881.

Research Question and PICOS Framework

The primary objective of the review was to evaluate and compare the marginal fit of dental copings fabricated from zirconia and PEEK using CAD/CAM technology. The research question was framed as: “Does the type of material (PEEK or Zirconia) influence the marginal fit of copings produced with CAD/CAM technology?”

Eligibility Criteria

To maintain methodological consistency and relevance, this review applied clearly defined inclusion and exclusion criteria, guided by the PICOS (population, intervention, comparison, outcome, and study design) framework, as outlined in Table 1. Studies were eligible for inclusion if they were in vitro experimental investigations assessing the marginal fit of full-coverage copings fabricated from both zirconia and PEEK using CAD/CAM technology. Accepted models included extracted human teeth, typodont models, or standardized dies, and studies were required to report marginal gap measurements quantitatively (e.g., mean ± standard deviation).

**Table 1:** PICOS framework used for checking the eligiblity of studies in the present systematic review CAD-CAM: Computer-aided design/computer-aided milling; PEEK: Polyethyletherketone; SD: Standard deviation

PICOS Component	Description	Inclusion Criteria	Exclusion Criteria
Population	Copings used in dental restorations	In-vitro studies involving zirconia and PEEK full-coverage copings on extracted human teeth, typodont models, or standardized dies	Animal studies, clinical trials, case reports, reviews, editorials
Intervention	PEEK copings fabricated using CAD/CAM technology	Studies evaluating CAD/CAM fabricated PEEK copings	Studies not using CAD/CAM fabrication for PEEK or evaluating PEEK in removable prostheses
Comparison	Zirconia copings fabricated using CAD/CAM technology	Studies directly comparing marginal fit of PEEK and zirconia copings	Studies evaluating only one material (PEEK or zirconia), or comparisons with materials other than zirconia
Outcome	Marginal fit accuracy (measured in µm)	Studies that report marginal fit values (mean ± SD) for both PEEK and zirconia copings using quantitative measurement techniques	Studies not reporting quantitative marginal fit measurements; studies without outcome-specific data
Study Design	In-vitro experimental studies	Laboratory-based in-vitro studies evaluating marginal fit of copings with appropriate measurement and statistical analysis	Clinical studies, pilot studies without full data, studies lacking control groups or valid statistical comparison

Only full-text articles published in English were considered. Studies were excluded if they involved materials other than zirconia or PEEK, did not provide direct comparisons between the two, or failed to report measurable marginal fit outcomes. Additional exclusions included clinical studies, animal experiments, case reports, editorials, reviews, and pilot studies with incomplete or inadequate data. Furthermore, studies involving compromised tooth structures (e.g., carious, restored, hypoplastic, or traumatically altered teeth) were omitted to avoid confounding variables affecting marginal adaptation.

Search Strategy

A comprehensive electronic literature search was carried out in multiple databases, including PubMed, SCOPUS, Web of Science, Google Scholar, Embase, and EBSCOHost, to identify relevant articles published between 2016 and 2025. No publication status or language restrictions were initially applied during the screening phase. The search strategy incorporated the keywords and MeSH terms “Zirconia”, “PEEK”, “Copings”, and “Marginal fit”, combined using Boolean operators. Manual cross-referencing of retrieved articles was also performed to capture additional eligible studies. The primary and alternate search strategies used in the PubMed database are provided in the appendices.

Study Selection Process

The study selection process was executed in two stages. Initially, two independent reviewers screened the titles and abstracts of all retrieved records against the eligibility criteria. Studies deemed potentially relevant were subjected to full-text review. In the second phase, the same reviewers independently assessed full-text articles for final inclusion. Disagreements between reviewers were resolved through mutual discussion and, if necessary, by involving a third reviewer acting as an arbitrator.

Data Extraction

Data extraction was performed using a predesigned, standardized extraction form. Two reviewers independently recorded relevant information, including author(s) and publication year, country of origin, study design, sample size, type and source of material (zirconia or PEEK), CAD/CAM system and milling protocols used, measurement techniques, number of points evaluated, mean marginal fit values, and statistical significance. Any inconsistencies or missing data were resolved by consensus or by contacting study authors when feasible.

Risk of Bias Assessment

Risk of bias in the included studies was evaluated using the QUIN tool (Quality Assessment Tool for In Vitro Studies), which is designed explicitly for laboratory-based experimental research [31]. Each study was assessed across nine domains: clarity of study aim, sample size justification, standardization of sample preparation, randomization of samples, blinding of outcome assessors, consistency in operator handling, outcome measurement method, statistical analysis reporting, and acknowledgment of study limitations. Each domain was scored as "Yes", "No", or "Partially", and an overall judgment was assigned as "Low", "Moderate", or "High" risk of bias. Risk assessments were independently conducted by two reviewers, with consensus reached through discussion in case of any discrepancies.

Data Synthesis

Narrative synthesis was employed to summarize and compare results where meta-analysis was not applicable due to methodological heterogeneity. Key outcome measures included marginal gap values in micrometers (µm), evaluated using stereomicroscopy, field emission scanning electron microscopy (FE-SEM), or digital image analysis software such as ImageJ or Leica Application Suite. Studies that reported sufficient statistical data (mean, standard deviation, and sample size) and followed comparable methodologies were pooled together for quantitative meta-analysis.

Statistical Analysis

Meta-analysis was performed using Review Manager (RevMan) software (Cochrane Collaboration, London, UK), where applicable. Standardized mean differences (SMDs) were calculated for marginal fit comparisons between PEEK and zirconia groups. Heterogeneity among studies was assessed using the I² statistic and the chi-square test. A fixed-effect model was used when heterogeneity was low (I² < 50%), and a random-effect model was employed when heterogeneity exceeded 50%. Sensitivity analyses were planned to explore the robustness of results by excluding studies with high or moderate risk of bias. Publication bias was evaluated through funnel plot analysis, with asymmetry interpreted in light of potential methodological differences.

Results

Study Locations and Design

**Figure 1:**
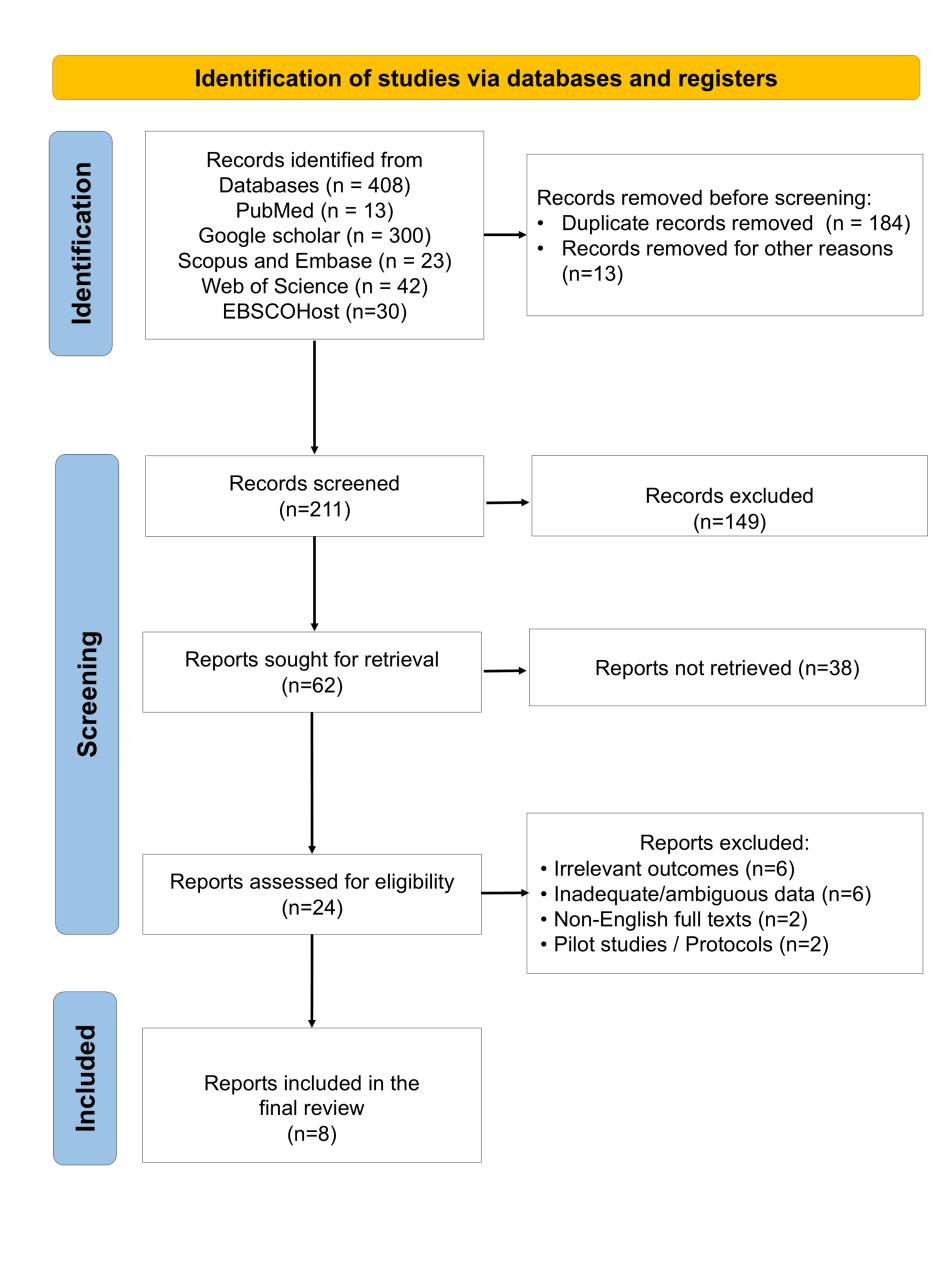
PRISMA flow diagram depicting the article selection process in the present systematic review PRISMA: Preferred Reporting Items for Systematic Reviews and Meta-Analyses

A total of eight in vitro studies published between 2019 and 2025 were included in this review (Figure 1) [32-39]. The data extracted regarding the study characteristics and outcomes are summarized in Table 2. These studies were geographically distributed between India (n=4) and Egypt (n=4). All studies employed standardized in vitro experimental designs, allowing controlled evaluation of marginal fit in dental copings.

**Table 2:** Data extracted from the studies included in the present systematic review PEEK: Polyetheretherketone; CAD: Computer-Aided Design; CAM: Computer-Aided Manufacturing; µm: Micrometer; SD: Standard Deviation; ANOVA: Analysis of Variance; HSD: Honestly Significant Difference; FESEM: Field Scanning Electron Microscope; SEM: Scanning Electron Microscope; NIH: National Institutes of Health; DWOS: Dental Wings Operating System; DWX: Dental Milling Unit Series (Roland DG); 3D: Three-Dimensional; K5: vhf camfacture AG 5-Axis Milling Machine; U200: Universal Self-Adhesive Resin Cement; RAY FOSTER: Milling/Model Fabrication System (USA); LAS: Leica Application Suite; ImageJ: Image Processing and Analysis Software (NIH, USA); µmm: Micrometer (measurement unit as reported); min: Minute; p: Probability Value; t: Student’s t-test; Zr: Zirconia; CAM System: Computer-Aided Manufacturing System; Typodont: Simulated Dental Model; FESEM: Field Emission Scanning Electron Microscope; CAD-CAM: Computer-Aided Design and Computer-Aided Manufacturing; SD: Standard Deviation; RSD: Relative Standard Deviation; M: Mean; N/A: Not Applicable; DWX-51D: Roland Dental Milling Machine; DTK-Kleber: Dual-Cure Composite Adhesive Cement (Bredent, Germany)

Author(s)	Year of Publication	Country	Study Design	Sample Size	Material Investigated	CAD-CAM System Used	Milling Machine Used	Coping Thickness (mm)	Type of Tooth/Model Used	Margin Design	Cementation Protocol	Measurement Technique	Number of Measurement Points	Measurement Software	Calibration Protocol	Mean Marginal Gap (PEEK)	Mean Marginal Gap (Zirconia)	SD (separate column only for meta-analysis)	Statistical Test Used	Author's Conclusion	Limitations Noted by Authors
Attia (2019) [39]	2019	Egypt	In-vitro	40 (20 PEEK, 20 Zirconia)	PEEK (milled, pellets, granules) and Zirconia	Dental Wings (DWOS V.8)	SHERA Werkstoff-Technologie, Lemforde, GmbH	0.5	Stainless steel master die simulating second lower molar	Heavy chamfer finish line (1.0 mm width)	Not cemented for marginal accuracy evaluation	Digital microscope with image analysis software	4 per coping (mid-labial, mid-lingual, mid-distal, mid-mesial)	Image J 1.43U (National Institute of Health, USA)	Using a ruler as reference for system calibration	Milled: 45.22 ± 6.09 µm, Pellets: 72.38 ± 9.75 µm, Granules: 78.69 ± 10.7 µm	43.98±1.3 µm	PEEK (Milled: 6.09, Pellets: 9.75, Granules: 10.7), Zirconia: 1.3	One-way ANOVA followed by pair-wise Tukey HSD test (P < 0.05)	PEEK CAD/CAM copings had significantly (p<0.001) better marginal accuracy than pressed PEEK copings; zirconia copings showed the highest accuracy, but all were within the clinically acceptable range (120 µm).	In-vitro study limitations; clinical validation required, lack of oral environment simulation, no veneering layer included.
Emad et al. (2020) [36]	2020	Egypt	In-vitro	40 (20 PEEK, 20 Zirconia)	PEEK, Zirconia	CEREC, SHERA eco-scan	SHERA eco-mill 50	0.7	Extracted human molars	Deep Chamfer	Duo-link resin cement	Stereomicroscope	points on each aspect of each crown are measured (with total 20 points)	Leica Application Suite	Standardized reference points	49.88 ± 7.97 µm	18.39 ± 3.1 µmm	PEEK: 7.97, Zirconia: 3.1	Mann-Whitney, ANOVA	PEEK veneered with composite can be used intraorally for single crown restorations, but zirconia showed significantly lower marginal gap values (p<0.001).	Effect of different milling axes on accuracy needs further study.
Makky et al. (2020) [37]	2020	Egypt	In-vitro	21 (7 zirconia (Zr), 7 PEEK CAD, and 7 PEEK press)	PEEK, Zirconia	SHERA eco-scan	SHERA eco-mill 5X	Not Specified	Typodont mandibular first molar	Deep Chamfer	Not Cemented	3D analysis (Triple scan)	8 points per section (20 sections total)	Poly Works Innovmetric	Best-fit alignment	69 ± 10 µmm	60 ± 10 µmm	PEEK: 10, Zirconia: 10	ANOVA, Post-hoc Tukey	Zirconia copings had a significantly superior (p=0.04) marginal fit compared to PEEK copings; all materials were within clinically acceptable limits.	Variability in scanner accuracy; need for larger sample size.
Amalorpavam et al. (2021) [32]	2021	India	In-vitro	30 (15 PEEK, 15 Zirconia)	PEEK, Zirconia	Not mentioned	RAY FOSTER, USA)	Not mentioned	Maxillary first premolar	Shoulder finish line	Rely X U200 Self-Adhesive resin (3M, Germany), applied to internal surface and luted with finger pressure for 10 min	Field Scanning Electron Microscope (FESEM)	7 per sample (buccal, lingual, 1mm from margin, highest convex cuspal tip, highest concave central fossa)	Not specified	Not specified	30.3 ± 5.1 µm	50.26 ± 16.02 µm	PEEK: 5.1, Zirconia: 16.02	One-way ANOVA, Dunnet t-test	PEEK showed significantly (p<0.05) better marginal fit and internal adaptation than zirconia; both were within clinically acceptable range.	Use of acrylic models instead of natural teeth, need for clinical trials, influence of die spacer on fit should be explored further.
Chouksey et al. (2023) [33]	2023	India	In-vitro	40 (20 PEEK, 20 Zirconia)	PEEK, Zirconia	Roland DWX-50, USA	vhf camfacture AG, Germany (K5)	0.7	Freshly extracted maxillary central incisors	Heavy chamfer margin of 1 mm wide supra gingival	White silicone indicator paste for internal coping fit, finger pressure used for seating	Field Emission Scanning Electron Microscope (FESEM)	Four points per coping (buccal, lingual, mesial, distal)	Not specified	Not specified	33.99 µm	56.21 µm	PEEK: 8.81 µm, Zirconia: 15.07 µm	One-way ANOVA, Independent t-test	PEEK copings showed a significantly (p<0.001) better marginal adaptation compared to zirconia.	Limited by in vitro conditions, not reflecting clinical scenarios; variations in marginal gaps due to different fabrication technologies.
Emam et al. (2023) [38]	2023	Egypt	In-vitro	18 (6 Zirconia, 6 PEEK CAD, 6 PEEK Pressed)	Zirconia, PEEK CAD, PEEK Pressed	Exocad Dental CAD (exocad GmbH, Germany)	DWX-51D (Roland DG, Australia)	0.5	Mandibular first molar typodont tooth	1 mm chamfer margin	Dual-cure composite adhesive cement (DTK-Kleber, Bredent, Germany)	Handheld digital microscope (40X magnification) & Image analysis software (ImageJ 1.53t, NIH, USA)	20 points per sample	ImageJ 1.53t (NIH, USA)	Calibrated before measurements	108.00 ± 20.08 µm (PEEK CAD), 108.00 ± 25.10 µm (PEEK Pressed)	48.67 ± 11.98 µm	PEEK CAD: ±20.08 µm, PEEK Pressed: ±25.10 µm, Zirconia: ±11.98 µm	One-way ANOVA, Tukey’s post hoc test, Welch one-way ANOVA	Zirconia copings exhibited significantly (p<0.001) better marginal adaptation than PEEK, but all materials had clinically acceptable values.	In vitro design does not replicate oral conditions; absence of thermocycling or aging; limited sample size.
Mahajan et al. (2024) [34]	2024	India	In-vitro	20 (10 PEEK, 10 Zirconia)	PEEK and Zirconia	exocad	Arum 5X-300 D milling machine	0.5	Extracted maxillary first premolars / 3D printed resin dies	Shoulder finish line	Self-adhesive translucent resin cement with finger pressure	Stereomicroscope at 60x magnification	3 points each on palatal and buccal sides (total 6)	Not specified	Not specified	Palatal: 23.941 µm, Buccal: 22.338 µm	Palatal: 18.469 Î¼m, Buccal: 17.116 Î¼m	PEEK: Palatal 7.73, Buccal 7.89; Zirconia: Palatal 6.55, Buccal 6.34	Two-way ANOVA and independent t-test	Zirconia showed better marginal fit (p<0.05) while PEEK had better fracture resistance	In-vitro study; resin dies used instead of natural teeth; no mechanical loading during cementation; stereomicroscope used instead of replica method
Jangade et al. (2025) [35]	2025	India	In-vitro	30 (15 PEEK, 15 Zirconia)	PEEK, Zirconia	SHINING 3D Software	4-axis milling unit	Not specified	Stainless steel master die, Type IV gypsum dies	360-degree rounded shoulder	None (dry fit)	Stereomicroscope with image analysis software	4 per coping	Geomagic Verify (Version 5.2)	Not specified	92.84 ± 3.48 µm	63.12 ± 31.47 µm	PEEK: ±3.48 µm, Zirconia: ±31.47 µm	Student's t-test	Zirconia exhibited superior marginal accuracy (t = 3.635, p = 0.001) compared to PEEK; both materials within clinically acceptable limits (<120 µm).	In-vitro study, lack of aging simulation, no internal fit evaluation.

Sample Size and Models Used

The included studies featured sample sizes ranging from 18 to 40 specimens, with most (n=6) dividing samples equally between zirconia and PEEK copings. Half of the studies (n=4) used extracted human teeth, such as maxillary incisors, premolars, and molars, offering anatomical fidelity that closely replicates clinical conditions [32-34,36]. The remaining studies (n=4) employed typodont models or synthetic dies, including resin or stainless steel substrates, which provided dimensional standardization but lacked the biomechanical complexity of natural teeth [35,37-39]. This variability in substrate choice introduces heterogeneity in marginal fit assessments, warranting caution when comparing findings across different model types.

CAD/CAM Systems and Milling Techniques

**Figure 2:**
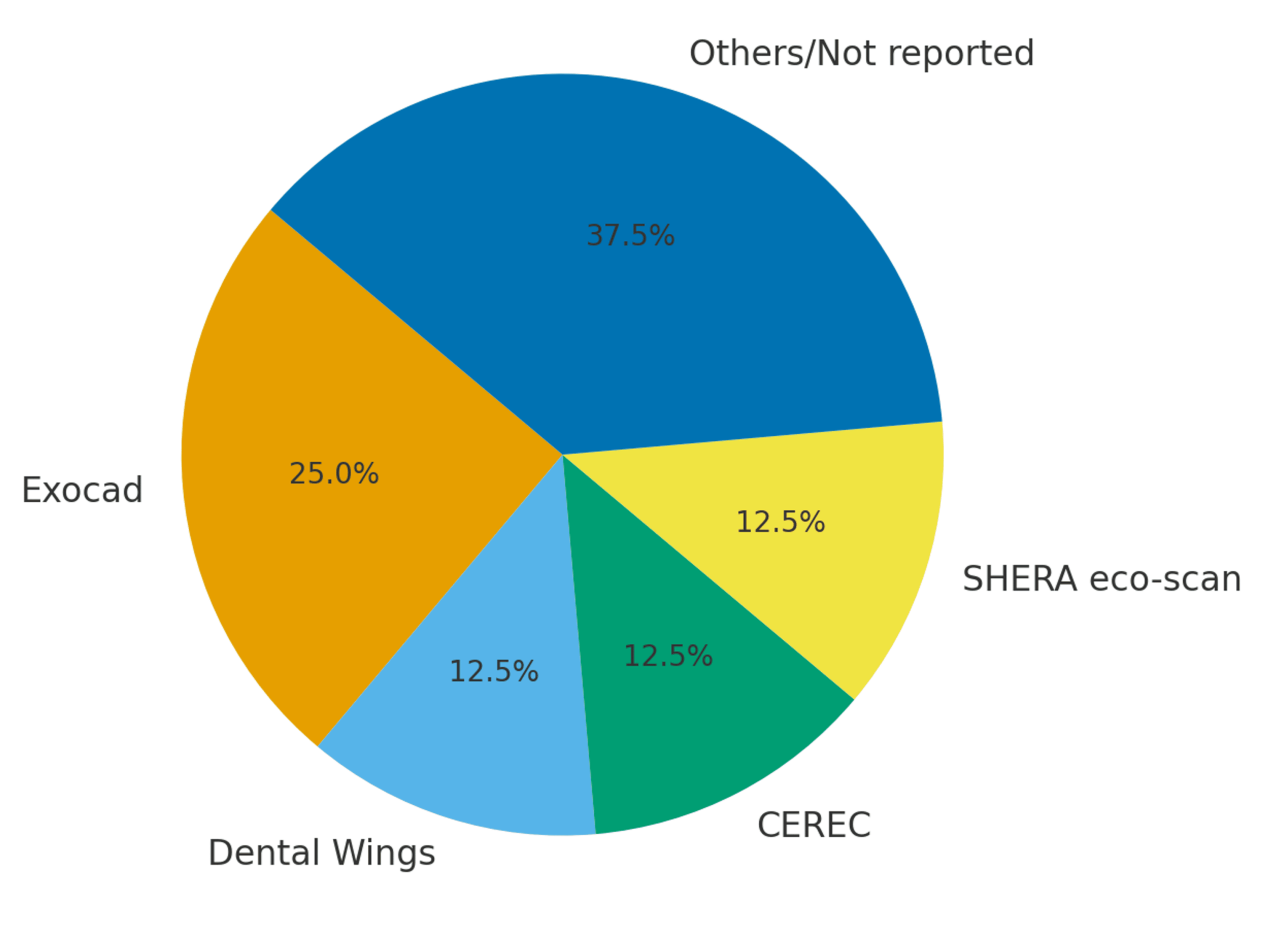
CADCAM softwares used for digital designing of the prosthesis across the included studies CAD/CAM: Computer-Aided Design and Computer-Aided Manufacturing

A variety of CAD design platforms were utilized across the studies (Figure 2). Exocad software was implemented in two studies [34,38], while Dental Wings, CEREC, and SHERA eco-scan were used in others [36,37,39]. These systems differ in their scanning resolution and margin-detection algorithms, potentially influencing coping accuracy. Milling units also varied, including advanced five-axis systems such as Roland DWX, VHF Camfacture AG, Arum 5X-300 D, and SHERA eco-mill. Most studies maintained coping thicknesses of 0.5-0.7 mm and used either a chamfer or shoulder finish line design, both of which are known to promote favorable marginal adaptation for rigid materials such as zirconia and PEEK.

Measurement Techniques and Calibration

All studies employed high-resolution imaging to assess marginal adaptation. FE-SEM was used in two investigations [32,33], while stereomicroscopy was used in two other investigations [34,36]. Image analysis software included ImageJ, Leica Application Suite, and PolyWorks InnovMetric, providing digital calibration for accurate, reproducible measurements [36-38]. The number of marginal measurement points per sample ranged from 4 to 20, typically distributed across buccal, lingual, mesial, and distal surfaces to capture a comprehensive profile of adaptation.

Marginal Fit Results

Reported marginal gap values varied across studies. Zirconia copings demonstrated the most minor gaps in several cases (e.g., 43.98 ± 1.3 µm, 18.39 ± 3.1 µm, and 60 ± 10 µm), often significantly outperforming PEEK alternatives [36,37,39]. However, two studies reported superior adaptation for PEEK, with values of 30.3 ± 5.1 µm and 33.99 ± 8.81 µm, compared to larger gaps in zirconia groups [32,33]. In studies evaluating multiple PEEK processing forms, CAD-milled PEEK consistently showed better fit than pressed or granular variants [38]. Overall, zirconia exhibited better adaptation in six studies [34-39], while PEEK was favored in two studies [32,33].

Statistical Analyses

All eight studies employed ANOVA for intergroup comparison, complemented by post hoc analyses such as Tukey’s or Games-Howell tests. The significance threshold was consistently set at p < 0.05. In terms of comparative outcomes, six studies (n=6) reported statistically better marginal fit for zirconia [34-39], while two (n=2) indicated superior or comparable performance by PEEK [32,33]. This statistical distribution reinforces the generally higher consistency and dimensional accuracy of zirconia, albeit with exceptions under optimized PEEK processing workflows.

Limitations of the Included Studies

All studies were conducted in vitro (n=8), lacking oral conditions such as saliva, thermal cycling, or occlusal loading, thereby limiting clinical generalizability. No study included aging simulations such as thermocycling or fatigue loading. Some used artificial dies instead of natural teeth, which could affect marginal fit accuracy. Sample size calculation, randomization, and blinding were inconsistently reported. Chouksey et al. and Emad et al. lacked both blinding and randomization [33,36]. Internal fit was not assessed in any of the studies, and calibration or operator consistency was either missing or partially described.

Conclusions Reported by the Authors

Most studies agreed that both zirconia and PEEK copings achieved clinically acceptable marginal fits (below 120 µm). However, zirconia showed statistically superior performance in six studies [34-39]. PEEK copings, particularly those fabricated via CAD/CAM, demonstrated equal or better performance in two studies [32,33]. Authors generally endorsed zirconia for its superior fit and dimensional stability, while acknowledging the potential of PEEK due to its biocompatibility and mechanical resilience. Calls for further clinical research under real-world conditions were uniformly emphasized across all included literature.

Meta-analysis

**Figure 3:**
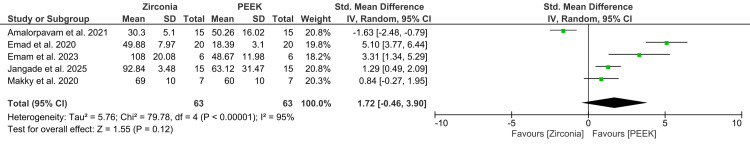
Forest plot showing the pooled results Source: Refs [32,35-38]

A meta-analysis of five studies (n=5), including 126 samples (63 per group), reported a pooled SMD of 1.72 µm (95% CI: -0.46 to 3.90 µm), favoring PEEK, though not statistically significant (Z = 1.55, p = 0.12). Heterogeneity was substantial (Tau² = 5.76, Chi² = 79.78, df = 4, p < 0.00001, I² = 95%). The pooled data of the meta-analysis concerning the marginal is represented as a forest plot in Figure 3.

**Figure 4:**
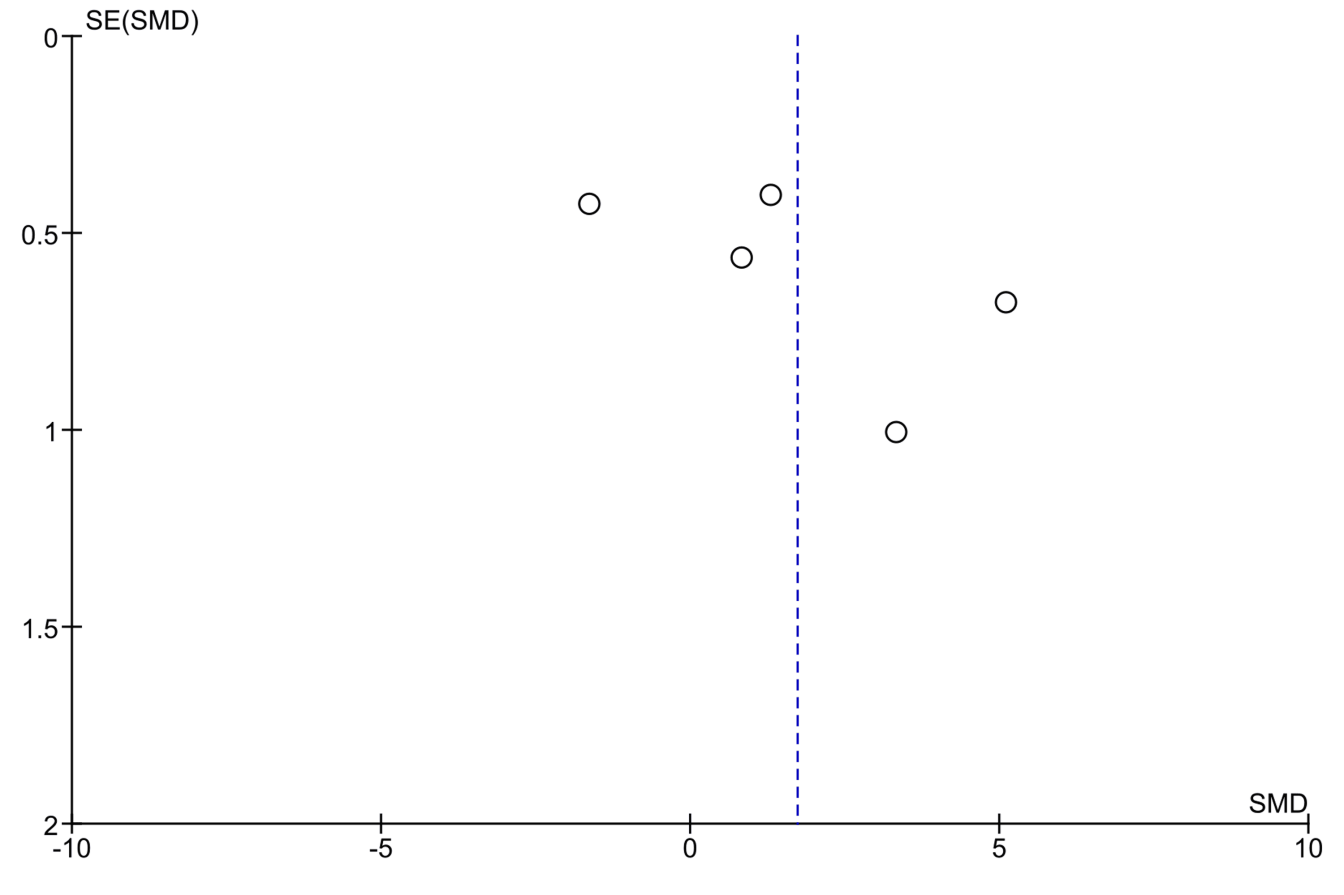
Funnel plot having minor asymmetry indicative of low publication bias Source: Refs [32,35-38]

The funnel plot showed no clear publication bias; minor asymmetry was attributed to methodological heterogeneity rather than selective reporting (Figure 4).

Risk of bias assessment

Among the eight studies, two were judged to have a low risk of bias based on their adherence to most QUIN domains (Table 3), including justification of sample size, standardization of procedures, and proper acknowledgment of study limitations [32,39]. The remaining six studies were categorized as having a moderate risk of bias [33-38]. These studies consistently reported well-defined study aims, standardized sample preparation, and appropriate outcome measurement methods. However, common methodological shortcomings included the absence of sample randomization and lack of blinding of outcome assessors, both of which may introduce performance or detection bias, particularly in high-resolution imaging studies where operator judgment can affect the measurement of marginal gaps.

**Table 3:** Risk of bias in the included studies according to QUIN tool

Study	Clarity of Aim	Sample Size Justification	Sample Preparation Standardization	Randomization of Samples	Blinding of Outcome Assessors	Consistency of Operator	Outcome Measurement Method	Statistical Reporting	Acknowledgment of Study Limitations	Overall Risk of Bias
Attia (2019) [39]	Yes	Yes	Yes	Partially	No	Yes	Yes	Yes	Yes	Low
Emad et al. (2020) [36]	Yes	Yes	Yes	No	No	Yes	Yes	Yes	Yes	Moderate
Makky et al. (2020) [37]	Yes	Partially	Yes	No	No	Yes	Yes	Yes	Yes	Moderate
Amalorpavam et al. (2021) [32]	Yes	Yes	Yes	No	Yes	Yes	Yes	Yes	Yes	Low
Chouksey et al. (2023) [33]	Yes	Yes	Yes	No	No	Yes	Yes	Yes	Yes	Moderate
Emam et al. (2023) [38]	Yes	Yes	Yes	Partially	Partially	Yes	Yes	Yes	Yes	Moderate
Mahajan et al. (2024) [34]	Yes	Yes	Yes	No	No	Yes	Yes	Yes	Yes	Moderate
Jangade et al. (2025) [35]	Yes	Yes	Yes	No	No	Yes	Yes	Yes	Yes	Moderate

While statistical analysis was adequately reported across all studies, and most studies acknowledged their limitations, the lack of randomization in six studies and the absence of assessor blinding in five highlight areas needing methodological reinforcement. Nevertheless, given the overall adherence to standardized in-vitro protocols, the collective quality of evidence remains moderate to high, supporting cautious yet valid synthesis of findings.

Discussion

The present systematic review evaluated eight in vitro studies published between 2019 and 2025 that compared the marginal fit of zirconia and PEEK copings fabricated using CAD/CAM technology [32-39]. Marginal adaptation is a critical parameter in fixed prosthodontics, as poor marginal fit can compromise the longevity of restorations by allowing microleakage, secondary caries, and periodontal inflammation [40]. All included studies used controlled in vitro designs, which enhance internal validity by eliminating biological variables but limit direct applicability to clinical conditions.

Across the studies, zirconia copings generally demonstrated superior marginal adaptation in six out of eight studies [34-39]. This finding aligns with the known advantages of zirconia, including dimensional stability, resistance to sintering shrinkage, and a high elastic modulus [41-43]. These properties facilitate precise milling and reduced distortion at the margins. In contrast, PEEK copings were reported to have better marginal adaptation in two studies [32,33]. These variations may be attributed to optimized milling parameters and the slight elastic flexibility of PEEK, which may allow it to better adapt to the die during seating, especially under low-load conditions [44,45].

While individual study results varied, all reported marginal gaps in both materials within clinically acceptable thresholds (<120 µm), reaffirming their suitability for clinical use. However, differences in CAD software, milling units, coping thickness, and tooth model substrates likely contributed to the variation in reported outcomes. For instance, advanced five-axis milling machines and high-resolution software platforms were associated with improved marginal precision, consistent with the literature [46-51].

The meta-analysis of five eligible studies revealed a pooled SMD favoring PEEK (SMD = 1.72 µm), but the result was not statistically significant (p = 0.12). The wide confidence interval (95% CI: -0.46 to 3.90 µm) and high heterogeneity (I² = 95%) indicate inconsistency across methodologies, including variation in measurement tools, scanning software, finish line designs, and substrate types. Despite this heterogeneity, funnel plot analysis showed no strong evidence of publication bias, suggesting balanced reporting of both favorable and unfavorable outcomes for each material.

Several limitations of the included studies must be acknowledged. None incorporated long-term clinical simulation through thermocycling, mechanical loading, or exposure to salivary enzymes, which are essential for approximating intraoral function over time [52-54]. The use of typodonts or resin dies in half of the studies further limits the generalizability of results, as these substrates do not replicate the elasticity and hydration of natural teeth. Methodological rigor was also variable, with randomization and blinding frequently omitted, and internal fit assessment neglected, which mainly limits the completeness of adaptation evaluation. Future studies should prioritize standardized protocols, inclusion of internal fit analysis, and simulation of clinical aging conditions to better inform material selection for long-term prosthetic success.

## Conclusions

Within the limitations of this systematic review and meta-analysis, it can be concluded that both zirconia and PEEK copings fabricated using CAD/CAM technology exhibit clinically acceptable marginal fit, reinforcing their suitability for fixed prosthodontic applications. Zirconia demonstrated a consistently superior marginal adaptation in the majority of included studies, likely due to its favorable mechanical and dimensional stability properties, while PEEK, particularly in CAD-milled form, showed comparable performance under optimized conditions. However, the substantial heterogeneity across studies, absence of intraoral simulation, and limited internal fit assessment highlight the need for further standardized, well-controlled clinical investigations. Future research should incorporate aging protocols, evaluate long-term performance under functional conditions, and explore material-specific CAD/CAM workflows to provide more definitive guidance for material selection in full-coverage restorations.

## Appendices

**Table 4:** Sample search string in the PubMed database

PubMed database	
Primary Strategy:	Search: (((Zirconia) AND (PEEK)) AND (Copings)) AND (Marginal Fit) ("zirconia s"[All Fields] OR "zirconias"[All Fields] OR "zirconium oxide"[Supplementary Concept] OR "zirconium oxide"[All Fields] OR "zirconia"[All Fields]) AND ("polyetheretherketone"[Supplementary Concept] OR "polyetheretherketone"[All Fields] OR "peek"[All Fields]) AND ("coped"[All Fields] OR "copes"[All Fields] OR "coping skills"[MeSH Terms] OR ("coping"[All Fields] AND "skills"[All Fields]) OR "coping skills"[All Fields] OR "coping"[All Fields] OR "copings"[All Fields]) AND (("margin"[All Fields] OR "margin s"[All Fields] OR "marginal"[All Fields] OR "marginals"[All Fields] OR "margined"[All Fields] OR "margins"[All Fields]) AND ("seizures"[MeSH Terms] OR "seizures"[All Fields] OR "fit"[All Fields]))
Translations	
Zirconia:	"zirconia's"[All Fields] OR "zirconias"[All Fields] OR "zirconium oxide"[Supplementary Concept] OR "zirconium oxide"[All Fields] OR "zirconia"[All Fields]
PEEK:	"polyetheretherketone"[Supplementary Concept] OR "polyetheretherketone"[All Fields] OR "peek"[All Fields]
Copings:	"coped"[All Fields] OR "copes"[All Fields] OR "coping skills"[MeSH Terms] OR ("coping"[All Fields] AND "skills"[All Fields]) OR "coping skills"[All Fields] OR "coping"[All Fields] OR "copings"[All Fields]
Marginal:	"margin"[All Fields] OR "margin's"[All Fields] OR "marginal"[All Fields] OR "marginals"[All Fields] OR "margined"[All Fields] OR "margins"[All Fields]
Fit:	"seizures"[MeSH Terms] OR "seizures"[All Fields] OR "fit"[All Fields]
